# UV-C mediated accumulation of pharmacologically significant phytochemicals under light regimes in in vitro culture of *Fagonia indica* (L.)

**DOI:** 10.1038/s41598-020-79896-6

**Published:** 2021-01-12

**Authors:** Bilal Haider Abbasi, Taimoor Khan, Razia Khurshid, Muhammad Nadeem, Samantha Drouet, Christophe Hano

**Affiliations:** 1 Department of Biotechnology, Quaid-i-Azam University, Islamabad, 45320 Pakistan; 2 Laboratoire de Biologie des Ligneux et des Grandes Cultures (LBLGC), INRA USC1328, Université ď Orléans, 45067 Orléans Cedex 2, France

**Keywords:** Biochemistry, Biotechnology, Plant sciences

## Abstract

*Fagonia indica* (L.) is an important medicinal plant with multitude of therapeutic potentials. Such application has been attributed to the presence of various pharmacological important phytochemicals. However, the inadequate biosynthesis of such metabolites in intact plants has hampered scalable production. Thus, herein, we have established an in vitro based elicitation strategy to enhance such metabolites in callus culture of *F. indica*. Cultures were exposed to various doses of UV radiation (UV-C) and grown in different photoperiod regimes and their impact was evaluated on biomass accumulation, biosynthesis of phytochemicals along antioxidant expression. Cultures grown under photoperiod (16L/8D h) after exposure to UV-C (5.4 kJ/m^2^) accumulated optimal biomass (438.3 g/L FW; 16.4 g/L DW), phenolics contents (TPC: 11.8 μgGAE/mg) and flavonoids contents (TFC: 4.05 μgQE/mg). Similarly, HPLC quantification revealed that total production (6.967 μg/mg DW) of phytochemicals wherein kaempferol (1.377 μg/mg DW), apigenin (1.057 μg/mg DW), myricetin (1.022 μg/mg DW) and isorhamnetin (1.022 μg/mg DW) were recorded highly accumulated compounds in cultures at UV-C (5.4 kJ/m^2^) dose than other UV-C radiations and light regimes.. The antioxidants activities examined as DPPH (92.8%), FRAP (182.3 µM TEAC) and ABTS (489.1 µM TEAC) were also recorded highly expressed by cultures under photoperiod after treatment with UV-C dose 5.4 kJ/m^2^. Moreover, same cultures also expressed maximum % inhibition towards phospholipase A2 (sPLA2: 35.8%), lipoxygenase (15-LOX: 43.3%) and cyclooxygenases (COX-1: 55.3% and COX-2: 39.9%) with 1.0-, 1.3-, 1.3- and 2.8-fold increased levels as compared with control, respectively. Hence, findings suggest that light and UV can synergistically improve the metabolism of *F. indica* and could be used to produce such valuable metabolites on commercial scale.

## Introduction

Plants have been conceded as enriched reservoir of pharmaceutically vital products due to wide spectrum of health attributes**.** Employing latest technological approaches, the focus of pharmaceutical industries is diverting towards specific compounds having respective metabolic role^1^. *Fagonia indica* (L.) is a remarkable pharmaceutical specie, belongs to the genus “*Fagonia*” and commonly known as “sacchi booti”^2^. This genus was named after Guy-Crescent Fagon, botanist and first physician to the French King Louis XIV (1638–1718). The species included in the genus *Fagonia* other than *Fagonia indica* are *Fagonia cretica, Fagonia olivieri* and *Fagonia Arabica*^3^. *Fagonia indica* is restricted to warm and arid zones of the world and can be found mostly in arid and semi-arid areas of Pakistan, India and other Asian areas globally^4^.

*Fagonia* species have been reported for having antibacterial and antifungal potential as well^5^. *F. indica* is being used traditionally for decades to treat asthma, dysentery, fever, urinary discharge, vomiting, leucoderma, typhoid while plant ash is used to treat anemia in children. The species has been cited as potential plant carrying great capacity to cure breast cancer^6,7^.

The pharmacological features of *F. indica* are authorized due to diverse phytochemicals constituents. An estimated range of compounds isolated and classified by employing this plant include phenolics, alkaloids, saponins, flavonoids, terpenoids^2^. The most widely occurring phytochemicals derived by secondary metabolism are phenolics, produced via phenylpropanoid pathway, pentose-phosphate pathway and shikimate pathway in plants^8^. Flavonoids comprise the biggest class of polyphenols in plants *i.e.* more than 8,000 were reported and the list is continuously expanding^9^. Phenolic compounds play vital aspects of developmental metabolism in plants, protection from harmful and pathogenic agents^10^. The above mentioned compounds are broadly distributed in plants; exist in specified organs, specific plants or distinct growth stages^11,12^. As phytomedicines; these compounds are significant and employed as antidiabetic, anti-inflammatory, anti-allergenic, hepatoprotective, antioxidant, antiatherogenic, anticancer, antimicrobial, vasodilatory agents, cardio-protective and antithrombotic^13^.

However, *its* medicinal preparations are formulated using plants collected from the wild; therefore, unrelated materials can be present in these commercial preparations. Recent innovative approaches based on tissue culture technology are alternatives for efficient production of phytochemicals instead of utilizing whole plants^14^. The concentrated production of metabolites has been praised broadly in literature via tissue culture techniques^15^. Being totipotent, almost each cell in the in vitro culture possesses whole genetic material and has the capacity for production of range of compounds than parental plants. Therefore, emerging trends of biotechnology and genetics mainly has emphasized to adopt in vitro culture techniques broadly in breeding and genetics, as model systems for plant pathology, physiology, biochemistry as well as biosynthesis medicinally vital secondary metabolites^16,17^. So, this technology is a substitute to synthesize the phytochemicals that are either sometimes difficult to obtain or expensive in terms of processing^18^. However, the lower contents, large fluctuations and inadequate processes are the key challenges associated with sustainable production from plants when utilizing in vitro culture technology^19^.

By employing the defense behavior of plants to stress, the innovative approaches towards the pharmaceutical vital plants production can be possibly established by optimizing conditions.

Elicitation is provoking such behavior of plants by induction of chemical defense as stress response; various types of physiological and molecular factors exist as elicitors^20^. They are basically classified into two groups as Biotic and Abiotic^2^. Elicitation is one among most widely used and cost effective approach for induction and to maximize the productivity of secondary metabolites by reducing time period as well as high yield in low volume of cultures^21,22^. However, the type, dose level and specificity of an elicitor along cultural conditions possess significant role in influencing elicitation^23^.

Among different approaches, the application of ultraviolet (UV) light as elicitor got more attention due to its tremendous effects on phytochemicals in several medicinal plant cultures^24,25^. The most effective type of UV irradiations proven yet in raising the plant metabolites production is UV-C (190–280 nm), employed in different vegetables, medicinal plants and fruits^26,27^. Mechanistically, the stress raised by UV light activates defense system in plants along producing phytoalexin^28^. The production of these compounds raise plant factories as more defensive responses including antioxidative enzymes, secondary metabolites and cell wall modifications, that cope the oxidative damage prompted by UV, through scavenging lethal reactive oxygen species (ROS)^29^. Other positive impacts include detained chlorophyll degradation, combating pathogens, and effects on nutritional features deeply studied by Shama and Alderson^30^ and Turtoi^31^. Alongside, recent developmental approach includes combinatorial use of elicitors for more efficient procedures and raised production of biomass. The application of different light regimes and UV-C has been proved to synergistically enhance production of ligans and neolignas in in vitro culture of *L. usitassium*^29^, and UV-C melatonin reported with significant effects by Nazir et al.^32^. For instance, the impacts of UV-C radiation along melatonin also affirmed on biosynthesis of anti-diabetic phytochemicals and antioxidants in in vitro cultures of *Lepidium sativum* L by Ullah et al.^33^.

Considering the stimulatory aspects of light elicitation and medicinal potential of *F. indica,* its derived in vitro cultures need to be more explored and further optimize its biomass assemblies and phytochemicals production. For this purpose, we have previously established protocol for biomass accumulation and pharmaceutically significant metabolites in callus cultures of *F. indica* applying elicitors^1,2^. In this sense, although the interest of these approaches is no longer to be demonstrated, our fundamental knowledge regarding production of *F. indica* biomass and phytochemicals under UV-C and different photoperiod regimes is missing. Currently, no previous report exists on elicitation by UV-C radiations for this species. Therefore, the current study aims to figure out the influence of UV-C radiations under various photoperiod regimes over biomass and phytochemicals accumulation in callus cultures of *F. indica*. Further, to assess the antioxidative profile via different in vitro cell free assays (DPPH, FRAP, ABTS) under UV-C stress and evaluate the biosynthesis of these compounds through HPLC. Moreover, to investigate the anti-inflammatory activities including sPLA2, 15-LOX, COX-1 and COX-2 in extracts derived from cultures exposed to UV-C and light regimes. So, this is the first study to show the interactive influence of light regimes and UV-C radiations on the accumulation of pharmacologically significant phytochemicals in in vitro culture of *F. indica* (L.).

## Results and discussion

### Combined effects of ultraviolet-C radiations and light regimes on biomass accumulation

**Figure 1:**
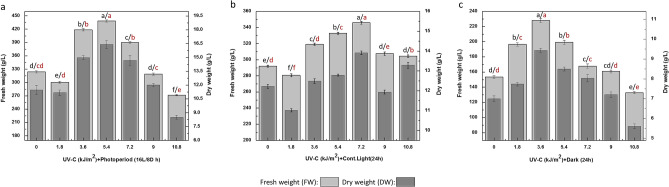
(**a**) Accumulation of Biomass in callus cultures of *Fagonia indica* under photoperiod (16L/8D h) after eliciting with various treatments (0–10.8 kJ/m^2^) of UV-C radiations. (**b**) Accumulation of biomass in callus cultures of *Fagonia indica* under contineous light (24 h) after eliciting with various treatments of UV-C radiations. (**c**) Accumulation of biomass in callus cultures of *Fagonia indica* under complete dark (24 h) after eliciting with various treatments (0–10.8 kJ/m^2^) of UV-C radiations. Values represented as mean ± SE of three replicates. Different (black for FW, red for DW) letters represent significant differences between the various experimental conditions (*p* < 0.05).

Light possess significant role in growth and development of plants. The application of different light sources as eliciting phytochemicals in pharmaceutical plants is getting deep attention. UV-C irradiation is one of them that play role in switching control expression of genes associated with growth and secondary metabolism of cells^34^. The current study involves the interactive influence of Ultraviolet-C radiation and different light regimes on callus cultures of *F. indica*. The results indicated optimal biomass accumulation (FW: 438.3 g/L; DW: 16.4 g/L) in cultures exposed to UV-C radiation (5.4 kJ/m^2^) for 30 min under photoperiod (16L/8D h) (Fig. 1a). This was followed by cultures grown under continuous light (24 h) with maximum biomass (FW: 345.9 g/L; DW: 13.9 g/L) at respective dose (7.2 kJ/m^2^) of UV-C radiation for 40 min and biomass accumulation (FW: 227.2 g/L; DW: 9.43 g/L) in dark after 20 min exposure to UV radiation (3.6 kJ/m^2^) as compared with controls respectively [Fig. 1b, c].

The UV-C radiations with growth proliferating effects have been reported for in vitro cultures of many medicinal plants previously^35,36^. The findings of current study show highest biomass accumulation among in vitro cultures of *F. indica* elicited previously^1,2^. Usually, a specified span of light is absorbed by photoreceptors in plants that are entailed in certain pathways of developmental processes. For instance, light act significantly in phytochrome diversions into far-red (active prf) and sustain genes expressions, resulting in raised cell division and metabolic activities as photo-response^1^. The higher biomass accumulation could also be resulted by induction of mutations in plant cells by UV-C light^24,25^ Furthermore; the photoperiod is an influencing factor responsible for growth and developmental processes in plants. Numerous studies revealed the predominant effects of light duration in regulating plant growth and levels of its significant metabolites in many plant species^37–39^. Carvalho et al.^40^; showed a dramatic increase growth and contents in *I. batatas* leaves by light irradiation for 16 h as compared to light exposure for short time.

Overall, the UV-C treatments for 20–40 min (1.2–7.2 kJ m^−2^) enhanced the callus proliferation but for extended duration of UV-C doses inhibited the cells growth under different photoperiod regimes.. The reduction in biomass accumulation at higher UV-C treatments could be the result of irreversible toxic effects to cells that might raise cell death in plant cultures^35,41^ and inducement of oxidative stress^42,43^. In addition, high light stress for extended hours induces photo-inhibition of photosynthetic system in plants. The resulting outcomes of extreme light exposures have previously mentioned by Kok^44^ and Lichtenthaler et al.^45^, those include imbalance in photosynthetic O_2_ and CO_2_ fixation, inhibition of chloroplasts and loosed fluorescence at PS II. Usually, photo inhibition occurs mostly by multifactorial light stress; distracting the QB proteins that is responsible for catalysis of QA (primary quencher) into heavy PQ assembly via electron transfer^46–48^. Furthermore, the decreased phenolics and flavonoids in cultures in continuous dark could also be due to degradation of chlorophyll contents resulting in reduced photosynthesis^49^.

Likewise, decreased cell growth and biomass proliferation in *Lepedium satvium* cultures under UV-C exposure for extended periods has been reported by Ullah et al.^33^. Our findings can be further elaborated and verified by several tissue cultures studies of pharmaceutical plants under UV-C exposure^36,50^.

**Figure 2:**
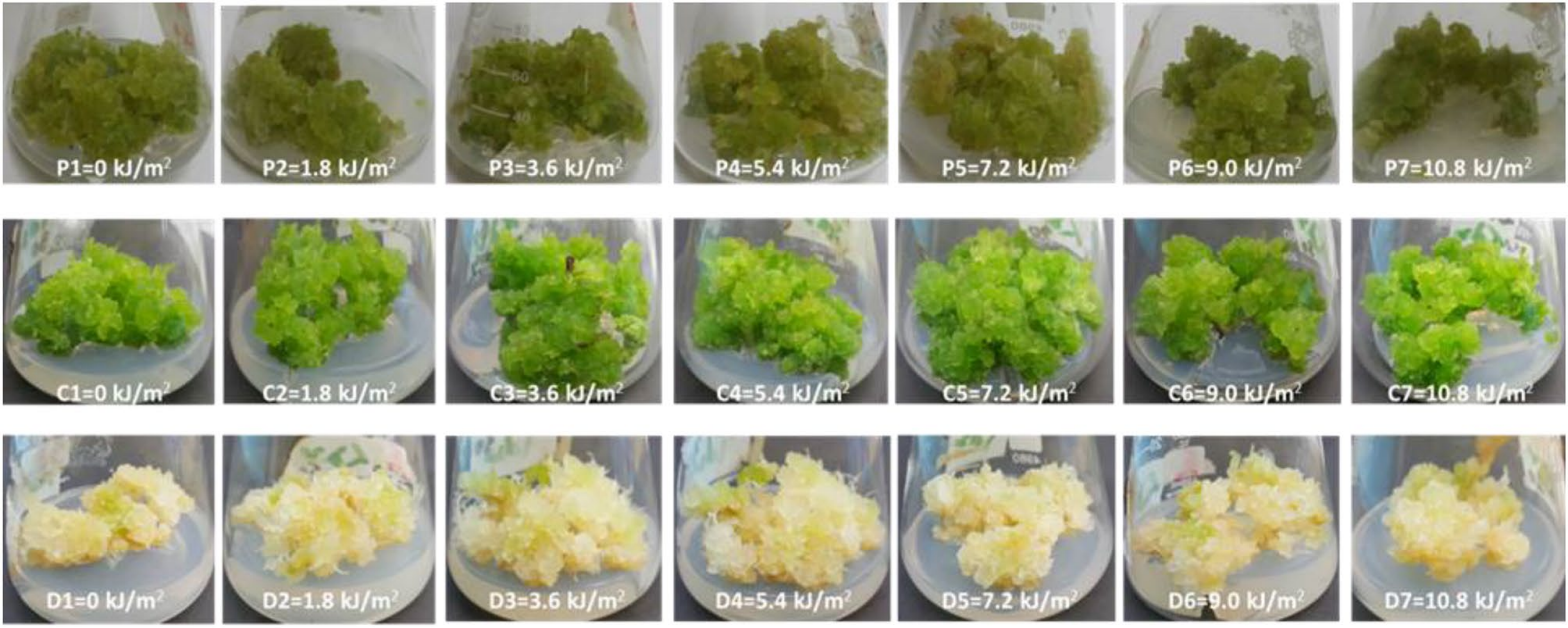
Combinatorial effects of UV-C treatments (0–10.8 kJ/m^2^) on morphology of callu cultures of *F. indica* grown for 30 days under light regimes; (**a**) P1–P2 stands for UV-C treated cultures in photoperiod (16L/8D h), (**b**) C1–C7 represents UV-C elicited cultures in cont. light (24 h) and (**c**) D1–D2 shows UV-C mediated cultures in complete dark (24 h).

Morphologically, all the cultures were found compact while calli under photoperiod (16L/8D h) were observed green, dark green under Cont. Light (24 h) and yellow-white in Dark (24 h) (Fig. 2). Naturally, the photosynthetic machinery in plants is adapted with a specific light/dark period. The extended dark phase is undoubtedly a significant stress factor for both the whole plant and photosynthetic system, resulting in reduced photosynthesis. For instance, prolonged dark period if continuously applied decrease the chlorophyll contents. Moreover, the possible reason could also be the senescence induced by both elicitors induced stress^49^.

The effects of UV-C may vary with the variations in dose, plant cells sensitivity and ability to vitiate the UV-C influence^51^. Contrarily, Khan et al.^3^ has reported maximum production of biomass in in vitro cultures of *F. indica* and in in vitro culture of *Linum usitatissimum* (L.) by Anjum, et al.^29^.

### Effects of UV-C radiations and various light regimes on total phenolic and total flavonoids accumulation

**Figure 3:**
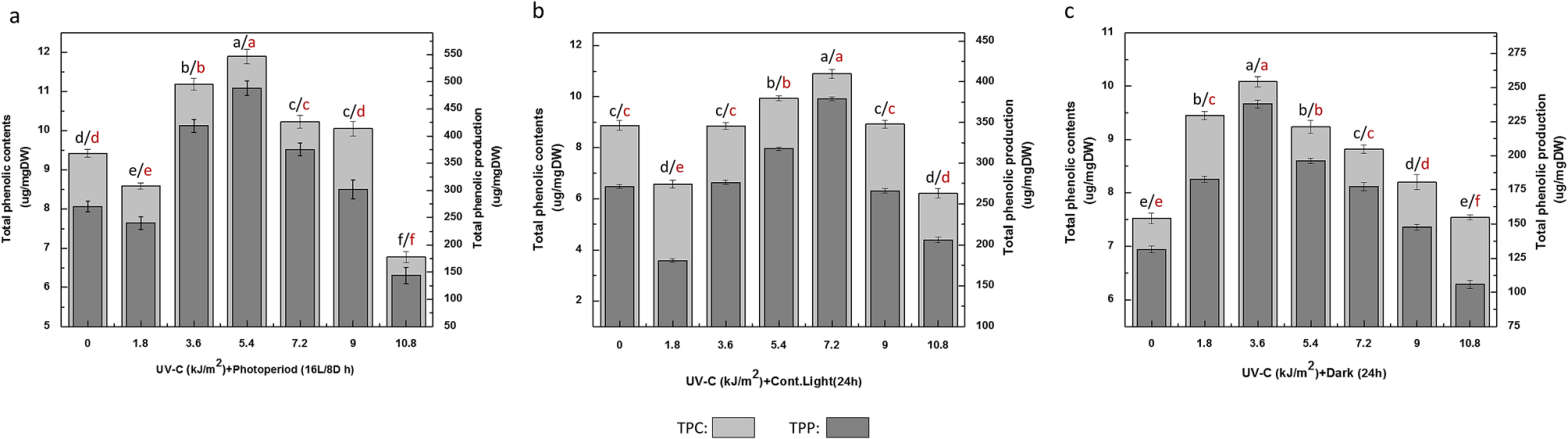
(**a**) Determination of Phenolic Contents in callus cultures of *Fagonia indica* elicited with various treatments (0–10.8 kJ/m^2^) UV-C radiations and grown in Photoperiod (16L/8D h). (**b**) Determination of Phenolic Contents in callus cultures of *Fagonia indica* elicited with various treatments (0–10.8 kJ/m^2^) of UV-C radiationsand grown in contineous light (24 h). (**c**) Determination of phenolic contents in callus cultures of *Fagonia indica* elicited with various treatments (0–10.8 kJ/m^2^) of UV-C radiationsand grown in complete darkness (24 h). Values represented as mean ± SE of three replicates. Different (black for TPC, red for TPP) letters represent significant differences between the various experimental conditions (*p* < 0.05).

Generally, plants receive various kinds of UV radiations that trigger diverse signal transduction pathways. This induces effects on secondary metabolism of plant species and eventually results in accumulation of distinct levels of plants metabolites^52^. In this study, the UV-C radiation and different photoperiod regimes were found with astonishing effects over biosynthesis of phenolic compounds in callus culture of *F. indica*. The cultures maintained under UV treatment associated with a photoperiod regime showed enhanced levels of total phenolics and total flavonoids contents than respective controls. However, the results indicated that optimum accumulation of phenolics (total phenolics contents (TPC): 11.8 μg GAE/mg DW, total phenolics production (TPP): 488.1 μg GAE/mg DW) was found in cultures under photoperiod (16L/8D h) at UV-C dose (5.4 kJ/m^2^) (Fig. 3a). This was followed by culture grown under continuous light (24 h) with the maximum levels of total phenolic contents (TPC: 10.8 μg GAE/mg DW; TPP: 378.2 μg GAE/mg DW) at UV-C dose (7.2 kJ/m^2^) in comparison to control [Fig. 3b]. Likewise, maximum production of total phenolics (TPC: 10.08 μg GAE/mg DW; TPP: 237.3 μg GAE/mg DW) was found in cultures in dark conditions at UV dose of 3.6 kJ/m^2^ (Fig. 3c).

Many studies exist about prompting effects of UV-C radiation over plant secondary metabolism^53,54^. The enhanced accumulation of flavonoids and phenolics under hermetic UV-C doses has been reported earlier in vegetables, fruits and other plant species^25,27^. However, the effect on secondary metabolites production in response to light exposure clearly depends not only on the considered species but also genetic variations among species^35,55^.

Although, earlier investigations reported about the signals transduction under UV irradiations, the exact mechanism of action behind UV-C effects is not properly known^56–58^. In the literature we can find some mechanistic opinions about influence of UV-C radiation over induction and enhancement of secondary metabolism of plants. Some studies indicate that a stimulation of the secondary metabolism under UV-C treatment can be triggered through the activation of key enzymes such as l-phenylalanine ammonia-lyase (PAL) and chalcone synthase (CHS). Such enzymes are responsible for catalyzing the main steps during biosynthetic pathways of many phenolics and flavonoids^28,59,60^.

**Figure 4:**
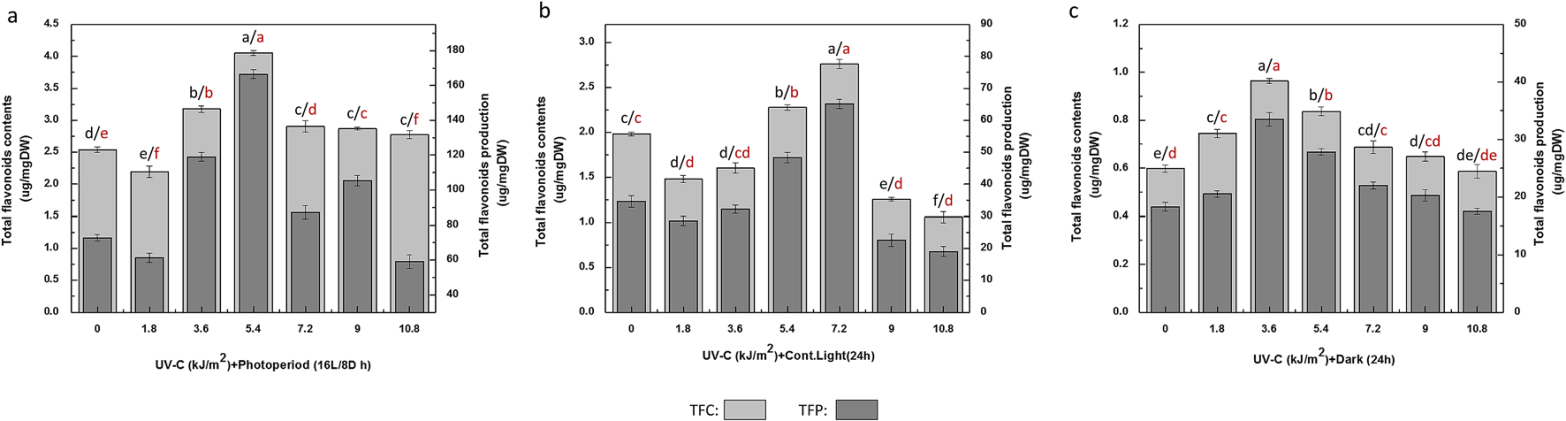
(**a**) Determination of flavonoids contents in callus cultures of *Fagonia indica* elicited with different doses (0–10.8 kJ/m^2^) of UV-C radiations and photoperiod (16L/8D h). (**b**) Determination of flavonoids contents in callus cultures of *Fagonia indica* elicited with different doses (0–10.8 kJ/m^2^) of UV-C radiations and contineous light (24 h). (**c**) Determination of flavonoids contents in callus cultures of *Fagonia indica* elicited with different doses (0–10.8 kJ/m^2^) of UV-C radiations under complete dark (24 h). Values represented as mean ± SE of three replicates. Different (black for TFC, red for TFP) letters represent significant differences between the various experimental conditions (*p* < 0.05).

Similar patterns were observed for total flavonoids contents and flavonoid production maintained under UV-C treatment associated with photoperiod regimes. The callus cultures maintained in photoperiod (16L/8D h) after UV-C (5.4 kJ/m^2^) treatment were found with optimum levels of flavonoids (total flavonoids contents (TFC): 4.05 μg quercetin equivalent (QE)/mg DW; total flavonoids production (TFP): 166.8 μg QE/mg DW), followed by UV-C treatment (7.2 kJ/m^2^) for 40 min in continuous light (24 h) with maximum flavonoids accumulation (TFC: 2.7 μg QE/mg DW; TFP: 65.09 μg QE/mg DW) respectively with comparative controls (Fig. 4a, b). However, the cultures grown in dark were found with low induction comparatively and maximum flavonoids accumulation (TFC: 0.96 μg QE/mg; TFP: 33.5 μg QE/mg DW) after exposure to UV dose (3.6 kJ/m^2^) as compared with control (Fig. 4c).

Flavonoids for considerable UV screening potential, the UV-C elicitation deeply enhance the flavonoids accumulation in response^61^. Notably, the flavonoids among all polyphenolics, play dominant role in plants defense against photodamages because of their considerable UV screening and antioxidant features^60,62–64^. Urban et al.^61^ reported that UV-C irradiations almost induce and raise flavonoids biosynthesis among all phenolic compounds. Moreover, Tiecher et al.^60^ found about the up regulation of CHS (chalcone synthase) and FLS (flavonol synthase) by UV-C radiation as key factors for flavonoid production^65^. The reduced levels of phenolics and flavonoids accumulation occurred at higher concentrations and fluctuating light exposures. The underlying mechanistic reasons include high levels of ROS generation, biological membranes disruption, photo inhibition, impairments in lipid metabolism, DNA damages and photosystem II destruction^66–68^.

However, influencing the biosynthesis of plants metabolites via UV-C treatment depends over elicitation levels as well, and increased levels can cause cells death in plants^41,69^. The same findings reported by Moon et al.^70^ in in vitro cultures of *Catharanthus roseus* (L.), where UV-C radiations enhance phenolics and flavonoids accumulation. Recently, comparative results on synergistic effects of UV-C and other elicitors have also been reported by Khan et al.^1^, Nazir et al.^32^ and Ullah et al.^33^.

### HPLC based quantification of phenolics and flavonoids

**Figure 5:**
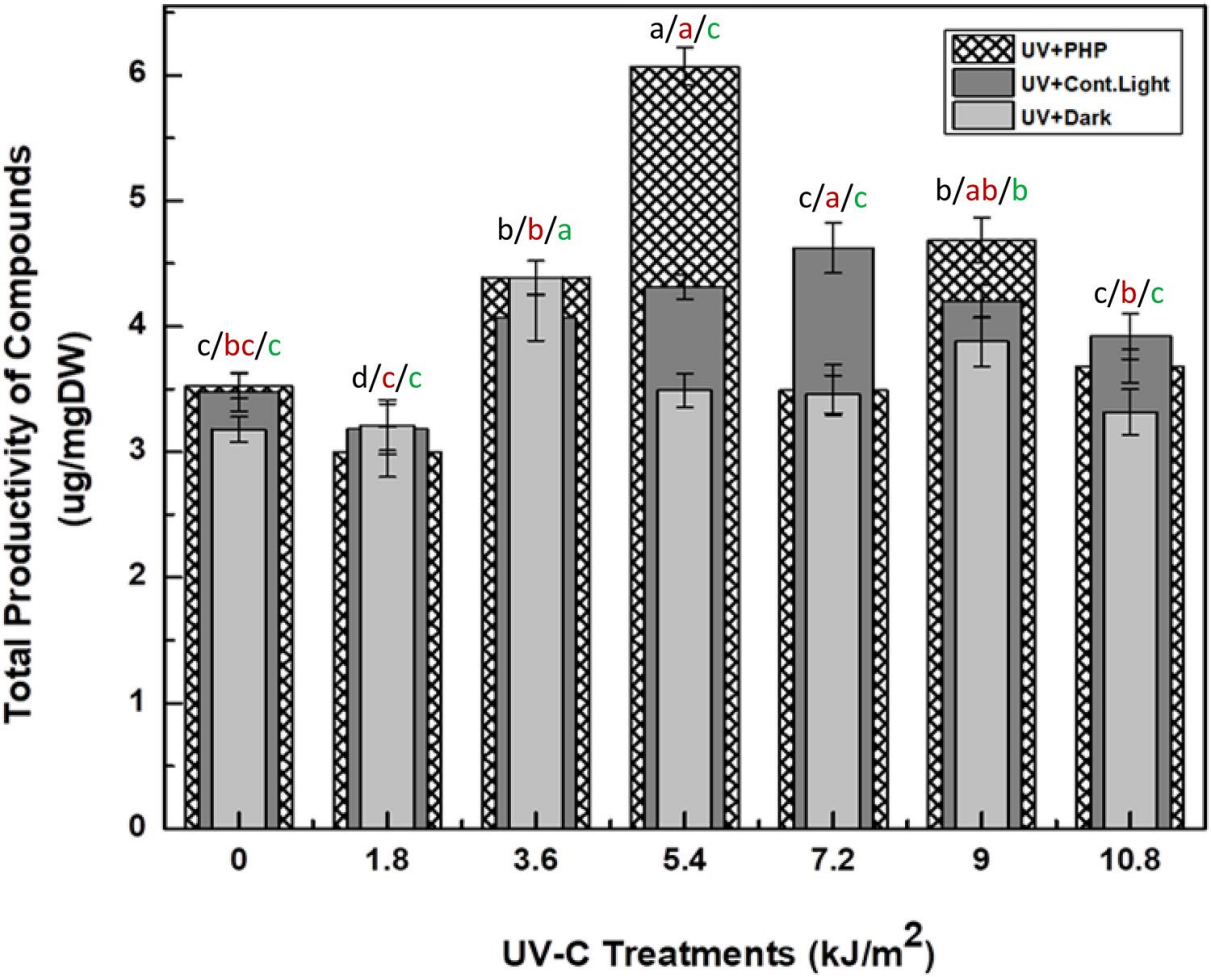
Productivity of 11 different phenolic compounds quantified via HPLC, in callus cultures of *Fagonia indica* after elicitation with UV-C radiations (0–10.8 kJ/m^2^) and maintained in different light regimes; cont. light (24 h), dark (24 h) and photoperiod (PHP, 16L/8D h). Values represented as mean ± SE of three replicates. Different (black for UV + PHP, red for UV + cont light, green for UV + dark) letters represent significant differences between the various experimental conditions (*p* < 0.05).

In this report, based on our previous reports^1,2^, a total eleven important phenolic metabolites have been evaluated in UV-C irradiated cultures under different photoperiod regimes via high performance liquid chromatography (HPLC). The HPLC evaluation showed that the highest accumulation of compounds (6.067 μg/mg ± 0.03) occurred in cultures grown in photoperiod (16L/8D h) after exposure to UV-C dose of 5.4 kJ/m^2^. The cultures treated with UV-C radiations (7.2 kJ/m^2^) for 40 min were found with total production of compounds (4.624 μg/mg ± 0.05) under continuous light (24 h) while least production (4.39 μg/mg ± 0.015) was found in cultures maintained in dark (24 h) after UV-C treatment (3.6 kJ/m^2^) as compared with control (Fig. 5).

UV-C irradiations have exerting damaging effects over plants including direct destruction of Plastoquinone (PQ) in chloroplast, disturbing activities in Mitochondria, DNA integrity and production of ROS and Peroxyl radicals formation^65^. A broad range of phenolics and flavonoids play role in reducing the photodamage by UV-C radiations due to screening of UV and antioxidative features^62–64,71^. Moreover, it is reported previously that the UV induces up regulation of CHS and FLS enzymes, activation of phytoalexins and several genes, decoding enzymes for biosynthesis of phenolics including PAL, CHS, anthocyanidin synthase (ANS) and stilbene synthase^61,72,73^.

The raised aggregation of compounds was noticed in cultures stressed with Ultraviolet-C treatments and various light regimes as compared with control (Table 1). Maximum levels of kaempferol (1.377 μg/mg DW), apigenin (1.057 μg/mg DW), myricetin (1.022 μg/mg DW) and isorhamnetin (1.022 μg/mg DW), followed by ursolic acid (0.235 μg/mg DW), nahagenin (0.226 μg/mg DW), hederagenin (0.207 μg/mg DW) and caffeic acid (0.175 μg/mg DW) were found in cultures prone to UV-C treatment (5.4 kJ/m^2^) subsequently maintained under photoperiod (16L/8D h) as compared with control. Likewise, the accumulation of gallic acid observed was (0.281 μg/mg DW), followed by betulinic acid (0.495 μg/mg DW) in cultures mediated with UV-C dose of 7.2 kJ/m^2^ under continuous light. Furthermore, the Catechins biosynthesis was recorded with optimum level (1.032 μg/mg DW) in cultures at UV-C dose of 3.6 kJ/m^2^ under dark conditions.

**Table 1:** HPLC based quantification of polyphenolic compounds in callus cultures of *Fagonia indica* elicited with UV-C doses (0–10.8 kJ/m^2^) under different photoperiod regimes.

	Gallic acid	Caffeic Acid	Myricetin	Catechin	Kaempferol	Isorhamnetin	Apigenin	Nahagenin	Hederagenin	Ursolic Acid	Betulinic Acid
**UV + photoperiod (16L/8D h)**											
UV (10.8 kJ/m^2^)	0.10 ± 0.001 d	0.08 ± 0.001 g	0.65 ± 0.002 i	0.52 ± 0.002 g	0.56 ± 0.001 i	0.65 ± 0.001 d	0.61 ± 0.002 e	0.23 ± 0.001 a	0.13 ± 0.01 c	0.09 ± 0.002 f	0.07 ± 0.002 l
UV (9.0 kJ/m^2^)	0.06 ± 0.002 f.	0.11 ± 0.003 d	0.78 ± 0.001 f.	0.63 ± 0.003 d	0.76 ± 0.001 d	1.0 ± 0.05 a	0.60 ± 0.002 f.	0.10 ± 0.01 e	0.11 ± 0.002 d	0.21 ± 0.001 c	0.30 ± 0.001 d
UV (7.2 kJ/m^2^)	0.05 ± 0.002 g	0.05 ± 0.002 j	0.63 ± 0.003 i	0.56 ± 0.001 f.	0.54 ± 0.002 ij	0.65 ± 0.001 d	0.57 ± 0.002 g	0.06 ± 0.004 h	0.12 ± 0.01 cd	0.07 ± 0.001 g	0.21 ± 0.002 g
UV (5.4 kJ/m^2^)	0.20 ± 0.002 b	0.18 ± 0.003 a	1.02 ± 0.04 a	0.72 ± 0.003 b	1.38 ± 0.03 a	0.82 ± 0.004 c	1.06 ± 0.02 a	0.19 ± 0.002 bc	0.09 ± 0.005 e	0.10 ± 0.001 e	0.26 ± 0.003 e
UV 3.6 kJ/m^2^)	0.18 ± 0.001 c	0.08 ± 0.001 g	0.92 ± 0.003 c	0.68 ± 0.002 c	0.56 ± 0.003 i	0.58 ± 0.003 e	0.77 ± 0.01 b	0.09 ± 0.002 f.	0.18 ± 0.003 b	0.09 ± 0.002 f.	0.25 ± 0.001 ef
UV (1.8 kJ/m^2^)	0.05 ± 0.001 g	0.05 ± 0.001 j	0.40 ± 0.003 m	0.47 ± 0.002 h	0.49 ± 0.003 k	0.52 ± 0.004 fg	0.44 ± 0.01 k	0.07 ± 0.01gh	0.21 ± 0.003 a	0.24 ± 0.001 a	0.06 ± 0.003 l
Control	0.10 ± 0.006 d	0.13 ± 0.001 c	0.61 ± 0.01 j	0.60 ± 0.005 e	0.49 ± 0.01 k	0.45 ± 0.003 hi	0.68 ± 0.002 c	0.08 ± 0.01 fg	0.06 ± 0.002 f.	0.23 ± 0.002 b	0.10 ± 0.003 j
**UV + cont. light (24 h)**											
UV (10.8 kJ/m^2^)	0.28 ± 0.01 a	0.15 ± 0.01 b	0.78 ± 0.01 ef	0.46 ± 0.01 h	0.63 ± 0.01 g	0.88 ± 0.01 b	0.54 ± 0.02 h	0.09 ± 0.001 f.	0.05 ± 0.005 f.	0.04 ± 0.001 h	0.09 ± 0.001 k
UV (9.0 kJ/m^2^)	0.07 ± 0.001 e	0.10 ± 0.02 e	0.85 ± 0.01 d	0.55 ± 0.01 f.	1.0 ± 0.01 b	0.45 ± 0.01 hi	0.67 ± 0.02 cd	0.19 ± 0.02 bc	0.03 ± 0.001 g	0.15 ± 0.001 d	0.15 ± 0.001 i
UV (7.2 kJ/m^2^)	0.07 ± 0.001 e	0.10 ± 0.01 e	0.96 ± 0.02 b	0.62 ± 0.01 d	1.0 ± 0.02 b	0.51 ± 0.01 g	0.53 ± 0.01 h	0.19 ± 0.01 b	0.03 ± 0.01 g	0.11 ± 0.01 e	0.47 ± 0.01 ab
UV 5.4 kJ/m^2^)	0.06 ± 0.001 f.	0.09 ± 0.003 f.	0.74 ± 0.01 g	0.69 ± 0.01 c	0.93 ± 0.02 c	0.54 ± 0.01 f.	0.55 ± 0.01 h	0.18 ± 0.01 bc	0.03 ± 0.001 g	0.07 ± 0.001 g	0.43 ± 0.01 b
UV (3.6 kJ/m^2^)	0.07 ± 0.001 e	0.10 ± 0.009 def	0.70 ± 0.01 h	0.54 ± 0.01 fg	0.67 ± 0.01 f.	0.48 ± 0.02 gh	0.64 ± 0.02 de	0.20 ± 0.02 b	0.03 ± 0.001 g	0.13 ± 0.02 de	0.49 ± 0.02 a
UV (1.8 kJ/m^2^)	0.06 ± 0.001 f.	0.08 ± 0.005 g	0.43 ± 0.01 l	0.47 ± 0.02 h	0.60 ± 0.01 h	0.39 ± 0.01 k	0.50 ± 0.03 h	0.15 ± 0.02 bc	0.03 ± 0.001 g	0.11 ± 0.01 e	0.37 ± 0.02 c
Control	0.07 ± 0.001 e	0.10 ± 0.001 e	0.71 ± 0.01 h	0.46 ± 0.01 h	0.46 ± 0.01 l	0.46 ± 0.02 hi	0.47 ± 0.02 ij	0.18 ± 0.01 bc	0.03 ± 0.001 g	0.11 ± 0.01 e	0.44 ± 0.02 b
**UV + dark (24 h)**											
UV (10.8 kJ/m^2^)	0.05 ± 0.001 g	0.07 ± 0.001 h	0.64 ± 0.01 i	0.69 ± 0.01 c	0.45 ± 0.01 l	0.48 ± 0.01 h	0.54 ± 0.01 h	0.09 ± 0.001 f.	0.03 ± 0.001 g	0.10 ± 0.01 ef	0.18 ± 0.01 h
UV (9.0 kJ/m^2^)	0.05 ± 0.001 g	0.07 ± 0.001 h	0.80 ± 0.01 e	0.62 ± 0.001 d	0.64 ± 0.01 g	0.51 ± 0.02 g	0.64 ± 0.02 de	0.12 ± 0.01 d	0.02 ± 0.001 gh	0.11 ± 0.01 e	0.29 ± 0.01 de
UV (7.2 kJ/m^2^)	0.04 ± 0.001 h	0.05 ± 0.001 j	0.81 ± 0.01 de	0.62 ± 0.01 de	0.50 ± 0.001 k	0.45 ± 0.01 i	0.55 ± 0.02 gh	0.10 ± 0.001 e	0.02 ± 0.001 gh	0.10 ± 0.001 e	0.23 ± 0.02 fg
UV (5.4 kJ/m^2^)	0.06 ± 0.001 f.	0.07 ± 0.001 h	0.75 ± 0.02 fg	0.48 ± 0.01 h	0.63 ± 0.02 fgh	0.43 ± 0.001 j	0.47 ± 0.001 j	0.15 ± 0.01 c	0.03 ± 0.001 g	0.06 ± 0.01 g	0.36 ± 0.01 c
UV (3.6 kJ/m^2^)	0.06 ± 0.001 f.	0.09 ± 0.009 ef	0.74 ± 0.02 fgh	1.03 ± 0.05 a	0.70 ± 0.01 e	0.50 ± 0.01 g	0.62 ± 0.01 e	0.15 ± 0.03 bc	0.03 ± 0.01 g	0.11 ± 0.05 e	0.36 ± 0.02 c
UV (1.8 kJ/m^2^)	0.05 ± 0.001 g	0.06 ± 0.001 i	0.61 ± 0.001 j	0.47 ± 0.01 h	0.54 ± 0.01 j	0.47 ± 0.01 h	0.49 ± 0.001 i	0.11 ± 0.03 cdef	0.02 ± 0.001 gh	0.10 ± 0.02 ef	0.27 ± 0.01 e
Control	0.06 ± 0.001 f.	0.08 ± 0.006 fg	0.52 ± 0.008 k	0.40 ± 0.01 i	0.52 ± 0.01 j	0.41 ± 0.01 jk	0.52 ± 0.02 h	0.15 ± 0.02 cd	0.03 ± 0.001 g	0.12 ± 0.01 e	0.35 ± 0.05 cd

Values are the mean of three triplicates ± standard error (SE). Different letters represent significant differences between the various experimental conditions (*p* < 0.05).

The usual hypothesis regarding UV-C radiations is same as other elicitors, raising the oxidative stress through the production of ROS^74^. ROS could have negative effects over cell components, only if not eliminated efficiently and accumulated. Prior to damage, the ROS play signaling role in networking physiological processes based on their redox status. The ROS engaged in induction and regulation of metabolic pathways affiliated secondary compounds biosynthesis^65^.

However, elicitation with UV-C radiation in various species usually occurred in PAL enzyme up-regulation during phenylpropanoid biosynthetic pathway^29^. Overall, our findings provide best feasible approach for optimum production of polyphenolics in UV-C elicited callus cultures of *F. indica* on both pilot and commercial scales. However, more studies would be needed to completely know biochemical and molecular mechanisms of production of these valuable phytochemicals under UV-C and light regimes treatments.

### Evaluation of antioxidants activity profile of *F. indica* callus cultures under UV-C radiations and photoperiod regimes

**Figure 6:**
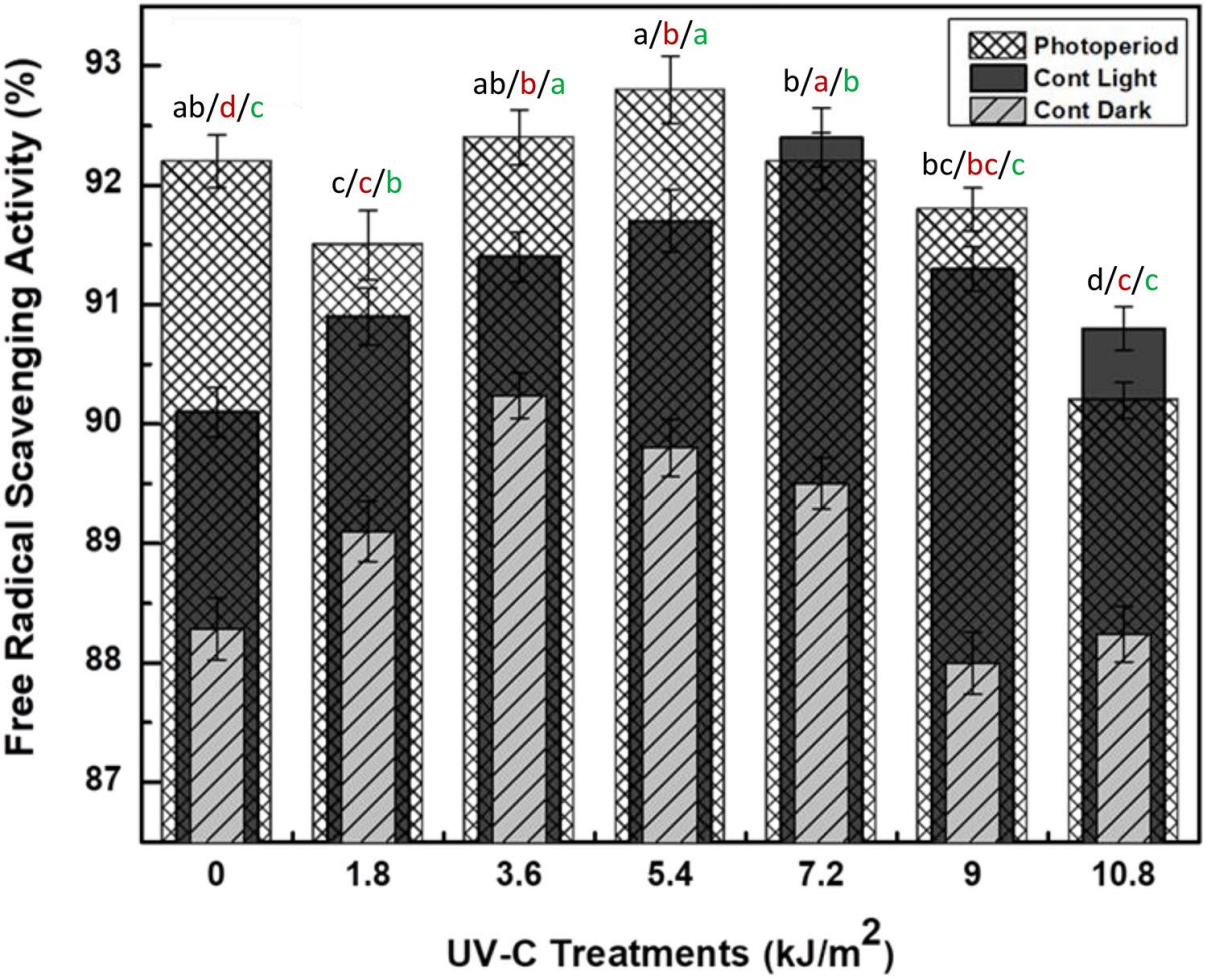
Free radical scavenging activity of UV-C radiations (0–10.8 kJ/m^2^) mediated callus cultures of *Fagonia indica* grown in different light regimes [cont. light (24 h), dark (24 h) and photoperiod (PHP, 16L/8D h)]. Values represented as mean ± SE of three replicates. Different (black for UV + PHP, red for UV + cont light, green for UV + dark) letters represent significant differences between the various experimental conditions (*p* < 0.05).

The callus cultures of *F. indica* irradiated by UV-C treatments under various photoperiod regimes were assessed for Antioxidant activity by employing DPPH, FRAP and ABTS assays. The DPPH assay is an extensively adopted technique to evaluate the antioxidant capacity of plant cells-derived products by considerable sensitivity, modest clarity and economic feasibility^75,76^. The results indicated; the optimum DPPH activity (92.8%) was recorded in UV-C (5.4 kJ/m^2^) mediated cultures maintained in photoperiod (16L/8D h) and DPPH activity (92.4%) in cultures elicited with UV-C (7.2 kJ/m^2^) for 40 min and grown in Continuous light (24 h) [Fig. 6]. The least DPPH activity (90.24%) was found in cultures exposed to UV-C dose of 3.6 kJ/m^2^ for 20 min and developed in dark conditions (24 h). Recently, Khan et al. reported the same correlation and phenolics based increase in DPPH activity in cell cultures of *F. indica* under continuous light^2^. In compliance with our findings, Erkan et al.^77^ and Li et al.^27^ have published a phenolic-dependent free radical scavenging activity (FRSA) after exposing the cultures of *Fragaria x ananassava* (Duchesne) to UV-C. The results conclusively show that rise in FRSA activity under UV-C treatments is because of enhanced accumulation of secondary phytochemicals as free radical scavengers^29^.

Likewise, the data showed that enhanced levels of FRAP and ABTS antioxidants activities were found in elicited cultures with UV-C radiations than controls (Table 2). Among different doses of UV-C radiations, the maximum FRAP antioxidant activity (182.32 µmol Trolox-C equivalent antioxidant capacity (TEAC)/ mg DW) was recorded at UV-C dose of 5.4 kJ/m^2^ applied to callus subsequently grown in photoperiod (16L/8D h), while cultures under continuous light (24 h) were found with FRAP activity (174.31 µmol TEAC/ mg DW) after exposure to UV-C (3.6 kJ/m^2^) for 20 mints. The cultures maintained in dark (24 h) after treating with UV-C doses showed the FRAP activity (194.27 µmol TEAC/ mg DW) at UV-C (7.2 kJ/ms^2^) as compared with control.

**Table 2:** Antioxidants potential activities of callus cultures of *Fagonia indica* elicited with UV-C radiations (0–10.8 kJ/m^2^) and grown in different photoperiod regimes using FRAP and ABTS cell free in vitro assays. Antioxidant capacity of samples was expressed in terms of TEAC (Trolox C equivalent antioxidant capacity) per mg of DW.

	FRAP	ABTS
**UV + photoperiod (16L/8D h)**		
UV (10.8 kJ/m^2^)	162.1 ± 0.6 f.	390.9 ± 0.1 h
UV (9.0 kJ/m^2^)	168.2 ± 0.4 e	474.4 ± 0.2 b
UV (7.2 kJ/m^2^)	177.7 ± 0.6 c	478.2 ± 0.6 b
UV (5.4 kJ/m^2^)	182.3 ± 0.6 b	489.1 ± 0.3 a
UV 3.6 kJ/m^2^)	179.8 ± 1.1 b	466.7 ± 0.4 c
UV (1.8 kJ/m^2^)	152.2 ± 0.6 g	425.2 ± 0.4 f.
Control	168.2 ± 0.6 e	412.1 ± 0.4 g
**UV + cont. light (24 h)**		
UV (10.8 kJ/m^2^)	158.5 ± 0.2 f.	456.4 ± 0.2 d
UV (9.0 kJ/m^2^)	160.2 ± 0.3 f.	472.2 ± 0.2 bc
UV (7.2 kJ/m^2^)	168.5 ± 0.4 e	486.1 ± 0.3 a
UV 5.4 kJ/m^2^)	155.4 ± 0.4 g	418.7 ± 0.1 f.
UV (3.6 kJ/m^2^)	174.3 ± 0.2 d	439.2 ± 0.4 e
UV (1.8 kJ/m^2^)	135.1 ± 0.4 i	304.6 ± 0.3 l
Control	162.4 ± 0.7 f.	384.9 ± 0.8 i
**UV + Dark (24 h)**		
UV (10.8 kJ/m^2^)	120.8 ± 0.3 k	380.8 ± 0.4 i
UV (9.0 kJ/m^2^)	125.0 ± 0.3 j	469.0 ± 0.3 c
UV (7.2 kJ/m^2^)	194.5 ± 0.2 a	444.7 ± 0.2 e
UV (5.4 kJ/m^2^)	126.5 ± 0.2 j	397.8 ± 0.4 h
UV (3.6 kJ/m^2^)	149.3 ± 0.2 g	442.3 ± 0.9 e
UV (1.8 kJ/m^2^)	109.2 ± 0.4 l	358.3 ± 0.2 j
Control	141.5 ± 0.4 h	341.8 ± 0.2 k

Values are the mean of three triplicates ± standard error (SE). Different letters represent significant differences between the various experimental conditions (*p* < 0.05).

Similarly, the trend was also noted down in ABTS activity. The data showed that maximum activity (489.11 µmol TAEC mg /DW) was recorded in calli mediated with UV-C dose (5.4 kJ/m^2^) and grown in photoperiod (16L/8D h) while cultures grown in continuous light (24 h) after 20 mints of exposure to UV-C radiations (7.2 kJ/m^2^) were found to enhance the ABTS activity up to (486.05 µmol TAEC mg /DW). However, the callus cultures under Dark (24 h) showed maximum activity (468.97 µmol TAEC mg /DW) after UV-C dose (9.0 kJ/m^2^) as compared with control.

A relationship can be established among antioxidants activities with production of biomass and phenolic contents in cultures exposed to UV-C radiations and maintained in various photoperiod regimes. The raised accumulation of phenolics and flavonoids could be the possible cause for raised antioxidant activities of grown calli under photoperiod at UV-C dose (30 min). Here, the highest significant correlation was obtained for myricetin and ABTS assay (Pearson Coefficient Correlation (PCC) = 0.935, *p* = 2.5 × 10^–5^) (Supplemental Table 1). Flavanoids were all significantly correlated with this antioxidant assay (Supplemental Table 1). The same correlation between phytochemicals and antioxidants potential has been stated previously^78,79^.

### Effects of UV-C radiations and Light regimes on Anti-inflammatory activities of *F. indica* callus cultures

Plant metabolites possess efficient enzyme inhibition feature that set off inflammatory activity in the body^80^. Inflammation happens as defense response against harmed cells, pathogens or any stimuli^81^. According various mechanisms reported for anti-inflammatory action such as inhibition of lipoxygenase (15-LOX, eicosanoid generating enzymes), cyclooxygenases (COX-1 and COX-2) and phospholipase A2 (sPLA2), dwindle the concentrations of prostanoid and leukotrienes^82^. The underlying phytochemistry behind strong antioxidant profile of *F. indica* explicates its anti-inflammatory features. Therefore, the current study also investigated the anti-inflammatory potential of *F. indica* callus cultures exposed to UV-C radiations and light regimes using in vitro cell free assays.

**Figure 7:**
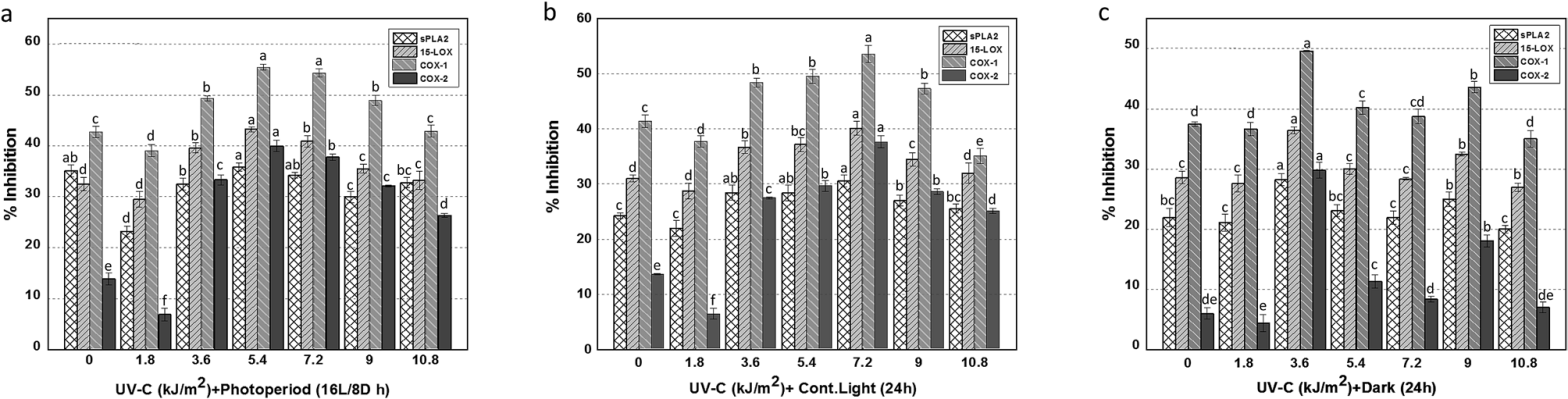
(**a**) Anti-inflammatory activities of callus cultures of *Fagonia indica* elicited with UV-C radiations (0–10.8 kJ/m^2^) and grown in photoperiod (16L/8D h) using sPLA2, 15-LOX, COX-1 and COX-2 cell free in vitro assays. The activities of extracts were expressed as % inhibition. (**b**) Anti-inflammatory activities of callus cultures of *Fagonia indica* elicited with UV-C radiations (0–10.8 kJ/m^2^) and grown in cont. light (24 h) using sPLA2, 15-LOX, COX-1 and COX-2 cell free in vitro assays. The activities of extracts were expressed as % inhibition. (**c**) Anti-inflammatory activities of callus cultures of *Fagonia indica* elicited with UV-C radiations (0–10.8 kJ/m^2^) and grown in complete dark (24 h) using sPLA2, 15-LOX, COX-1 and COX-2 cell free in vitro assays. The activities of extracts were expressed as % inhibition. Employed under the same experimental conditions as sample extracts, reference inhibitors were used as positive controls: Ibuprofen (10 µM) was used as positive control for COX-1 and COX-2 activity leading to enzyme inhibition of 31.4 ± 0.8% and 29.8 ± 1.2%, respectively; Thioetheramide-PC (5 µM) was used as sPLA2 inhibitor, resulting in an inhibition of 43.7 ± 0.8%; Nordihydroguaiaretic acid (100 µM) was used as 15-LOX inhibitor, leading to an inhibition of 30.6 ± 0.7%. The same volume of extraction solvent was used as blank. Values represented as mean ± SE of three replicates. Different letters represent significant differences between the various experimental conditions (*p* < 0.05).

Optimum % inhibition (sPLA2: 35.8%), 15-LOX: 43.3%), (COX-1:55.3%), and (COX-2: 39.9%) was found in cultures exposed to UV-C dose (5.4 kJ/m^2^) and grown under (16L/8D h) photoperiod as compared with control respectively (Fig. 7a). Whereas, the cultures treated with UV-C radiation (7.2 kJ/m^2^) and maintained under continuous light (24 h) expressed maximum inhibitory activities towards sPLA2 (28.1%), 15-LOX (36.4%), COX-1 (49.5%) and COX-2 (29.8%) as compared with control (Fig. 7b). For instance, optimum levels of sPLA2 (30.5%), 15-LOX (40.1%), COX-1 (53.4%), and COX-2 (37.5%) activities were recorded in cultures elicited with UV-C dose (3.6 kJ/m^2^) for 20 min and grown in dark conditions (24 h) (Fig. 7c). The highest significant correlation was obtained for kaempferol and isorhamnetin (Supplemental Table 1). Here, with the exception of apigenin and ursolic acid, all analyzed phytochemicals were significantly associated with potential anti-inflammatory action (Supplemental Table 1). It is the first report on documenting the anti-inflammatory activities of *F. indica* cultures treated with UV-C radiations and light regimes. Despite, the *F. indica* has previously been exploited for its anti-inflammatory potential and analgesics in mice^83^.

COXs are cyclooxygenases that are responsible for maintaining homeostasis in the kidney, produced during cancer and other inflammations. They are extensively used to study anti-inflammatory potentials of plant extracts because plants have natural phytochemical inhibition mechanisms that inhibit them^84^. The compounds detected possess strong anti-inflammatory potentials^85^.

## Conclusion

Conclusively, the current study involved the elicitation strategy via UV-C radiations under different photoperiod regimes in callus cultures of *Fagonia indica* (L.). The UV-C treatments effectively raised the production of phenolic compounds and biomass proliferation. The cultures exposed to UV-C radiations (5.4 kJ/m^2^) for 30 min and grown under photoperiod (16 h light /8 h dark) were found worthwhile sources of phytochemicals and biomass production. The highest levels of phytochemicals, anti-inflammatory and antioxidants activities were recorded on the same treatment of UV-C under photoperiod. Our data provide evidences showing that elicitation of *F. indica* callus cultures with UV-C radiation is a viable strategy for a safe, sustainable and enhanced production of biomass and phytochemicals. We anticipate that scale up studies at both pilot and commercial scales could confirm these trends. This will definitely assist with decoding the molecular mechanisms behind maximum expression of genes responsible for growth and phytochemicals production.

## Materials and methods

### In vitro seeds germination

The seeds of *Fagonia indica* (L.), were identified by a taxonomist at Quaid-i-Azam University, Islamabad. Seeds were sterilized and inoculated on MS-0 media according to the protocol of Ahmad, et al.^86^. Briefly, the Incubation Dry Separation Floating technique was applied to determine viable seed^87^. Briefly, autoclaved distilled water was used to rinse seeds, followed by treatment with HgCl2 (0.1%) and ethanol (70%) for a period of 2 min for surface sterilization of seeds. In order to remove impurities, seeds were entirely washed 3 times with autoclaved distilled water, followed by surface drying of seeds with autoclaved filter papers. The growth media (MS0; Murashige and Skoog basal medium; Phytotechnology Labs, USA)^88^ supplemented with sucrose (30 g/L) as carbon source and 8 g/L of agar (Phytotechnology Labs, USA) as solidifying agents was utilized for inoculation of seeds. The media was autoclaved (Systec VX 100, Germany) at 121 °C for 20 min after pH calibration (5.5–5.7; Eutech Instruments pH 510, Singapore) using NaOH (0.1 N) and HCL (1.0 N) to produce contamination free plantlets. The flasks having seeds inoculated on media were shifted to growth chamber and kept under fixed growth conditions i.e. temperature 25 ± 2 °C in photoperiod (16/8 h light/dark) and light intensity of 40–50 μmol m^−2^ s^−1^ (Philips TLD 35 Florescent lamps).

### Establishment and treatment of callus culture with UV-C and light regimes

The 30 days grown in vitro plantlets were used as source of explant. The stem explant (1 cm) was used for maximum callus biomass production as previously optimized by our research group^2^. Briefly, MS media fortified with sucrose (30 g/L), agar (8 g/L) and Thidiazuron (4.4 µM) with PH (5.5–5.7) was employed for inoculation of explants. The cultures maintained in conditions of (16L/8D hours) light and temperature 25 °C.

The experiment of UV-C elicitation was performed using 4 weeks grown calli by following the protocol of Anjum et al.^29^. The UV-C radiations were applied on cultures at distance of 15 cm from Ultraviolet-C lamp (254 nm, Model ZQJ-254, China with value (intensity) of 3 W/m^2^). The experimental Ultraviolet-C treatments (kJ/m^2^) were calculated as;

$$ {\text{UV-C dose}} = {\text{Intensity of Radiation }}\left( {{\text{W}}/{\text{m}}^{{2}} } \right) \times {\text{Exposure time of Cultures to UV-C}} $$

**Figure 8:**
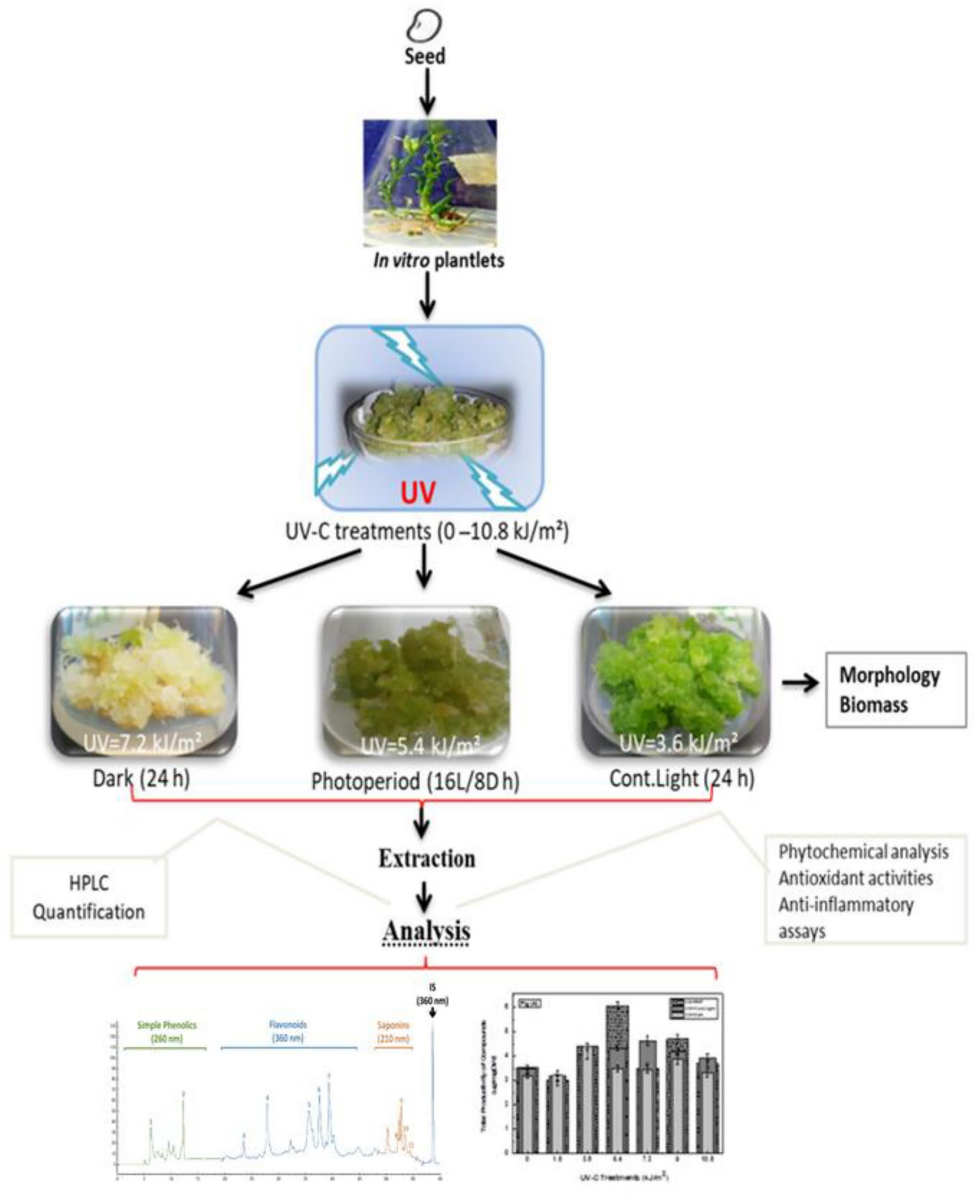
Graphical represenation of synergistic effects of UV-C treatments (0–10.8 kJ/m^2^) and light regimes (cont. light (24 h) and complete dark (24 h) on callus cultures of *Fagonia indica*.

Total 6 Ultraviolet-C doses 0, 10, 20, 30, 40, 50, and 60 min equivalent to 0, 1.8, 3.6, 5.4, 7.2, 9.0, and 10.8 kJ/m^2^ were used. First, the Ultraviolet-C lamp was made stabilized by leaving it ON for 15 min prior experiment. Then, fresh callus cultures were equally dispersed in sterile petri plates with sterilized forceps, to fully expose and properly irradiate with Ultraviolet-C doses (1.8–10.8 kJ/m^2^) for 10–60 min total duration. The callus cultures (0.5 g) were inoculated back into Erlenmeyer flask (100 ml) having fresh culture media (40 mL) after UV-C exposure. Subsequently, the cultures were moved to growth room (25 ± 2 °C) and maintained under their relevant sub-experimental conditions i.e.; complete 24 h-dark, 24 h-continuous light and 16L/8D h photoperiod. The light conditions in different light regimes were as; continuous light (24 h light) continuous dark (complete 24 h dark conditions) and photoperiod (16 h light and 8 h complete dark conditions). The cultures in each light regime without UV-C exposure (UV-C 0 kJ/m^2^) were used as respective controls. Three biological replicas were used in each sub-experiment for respective UV-C treatment. Finally, after 30 days of growth period, the callus cultures were harvested, followed by investigation of biomass and stored for further analysis. The different experimental steps of whole study are mentioned in a schematic diagram (Fig. 8).

### Determination of biomass and extract preparation

For Fresh weight investigation, the callus cultures were harvested and placed on filter paper to remove leftover water or media. The callus was subsequently weighed to determine Fresh weight biomass whereas for Dry weight calculation, the cultures were oven dried prior weighing.

For phenolic, antioxidants and anti-inflammatory activities determination, the methanolic extracts of cultures were prepared by using the protocol of Ali and Abbasi^75^ with little modifications. Briefly, the 10 mL of methanol (99.9%) was used to thoroughly dissolve 200 mg of each powdered callus (dried at 50 °C) and placed in rotatory for 24 h at room temperature. The reaction mixtures were then sonicated for 25 min (Toshiba, Japan) followed by vortexing for 5 min. The procedure for phytochemicals extraction was repeated twice and mixtures were then centrifuged at 6000 rpm for 15 min. The supernatants were evaporated to dryness before being subjected to cellulase R10 from *Trichoderma reesei* (Sigma Aldrich) digestion for aglycone release in citrate phosphate buffer (pH4.8) using an enzyme concentration of 2 units/mL during 6 h at 40 °C under agitation (200 rpm) in a water bath.

### Determination of phenolic and flavonoids contents

The determinations of total phenolic content (TPC) was performed via utilizing Folin- Ciocalteu (FC) reagent by the modified protocol of Arias, et al.^89^. Briefly, each sample (20 μL) extracted was mixed with 90 μL of the FC reagent in 96 well plate (10 × diluted with distilled water) followed by 5 min incubation at room temperature (25 ± 2 °C). Then, (6%, w/v) sodium carbonate (90 μl) was added to the wells, incubated for 90 min at room temperature. Gallic acid (1 mg/mL) and methanol (20 μL) were used as positive and negative controls respectively. The absorbance was noted at 725 nm with UV–Visible spectrophotometer (Shimadzu-1650; Japan). Gallic acid (0–40 μg/mL) was used as standard for plotting calibration curve (R^2^ = 0.967) and the TPC was expressed as gallic acid equivalents (GAE)/g of DW. Total phenolic production (TPP) was calculated by using the following formula and expressed in mg gallic acid equivalent/l.

$$ {\text{Total phenolic production mg/L}} = {\text{DW }}\left( {{\text{g}}/{\text{L}}} \right) \times {\text{TPC }}({\text{mg}}/{\text{g}}) $$

For total flavonoid content (TFC) determination, the aluminum chloride (AlCl_3_) colorimetric method was used as reported by Xu et al.^50^ with minor modifications. Briefly, 20 μL of the extracted sample was mixed with 10 μL of potassium acetate (1 M) and 10 μL of AlCl3 (10%, w/v). This mixture was incubated for 30 min at room temperature (25 ± 2 °C) after addition of 160 μL of distilled water to adjust the total volume to 200 µl. Absorbance of the reaction mixture was measured at 415 nm by using UV–Visible spectrophotometer (Shimadzu-1650; Japan). Quercetin (0–40 μg/mL) was used as standard for plotting calibration curve (R^2^ = 0.989) and the TFC was expressed as quercetin equivalents (QE)/g of DW. Total flavonoid production (TFP) was calculated by using the following formula and expressed in mg quercetin equivalent/l.

$$ {\text{Total flavonoid production mg/L}} = {\text{DW }}\left( {{\text{g}}/{\text{L}}} \right) \times {\text{TFC }}({\text{mg}}/{\text{g}}) $$

### Determination of antioxidant activities

The UV-C mediated cultured under various photoperiod regimes were analyzed for their respective level of antioxidants activities via DPPH, FRAP and ABTS assays.

- DPPH scavenging activity

To determine the antioxidant activity, 2, 2-diphenyl-1-picrylhydrazyl (DPPH) free radical scavenging assay (FRSA) was performed using the method narrated by Fazal et al.^55^ with slight modifications. Briefly, the sample extracts (20 μL) and 180 μL of DPPH (3.2 mg/100 mL methanol) were mixed in each well of 96 well plates, and then incubated in dark for 1 h at room temperature (25 ± 2 °C). Absorbance of the reaction mixture was measured at wavelength (517 nm) by using UV–Visible spectrophotometer (Shimadzu-1650; Japan). The final concentrations of ascorbic acid (10, 05, 40 and 20 μg/mL) and DMSO (20 μl) with DPPH (180 μL) were loaded as negative control. The radical scavenging activity was calculated as percentage of DPPH discoloration using the following equation;

$$ {\text{Free radical scavenging activity }}\% \, = \, 100 \times (1 - {\text{A}}_{{\text{E}}} /{\text{A}}_{{\text{D}}} ), $$

where A_E_ is absorbance of the solution when aculture extract was added at a particular concentration and A_D_ is the absorbance of the DPPH solution (standard).

- Ferric Reducing Antioxidant Power (FRAP) Assay

The FRAP potential of cultures was determined via method of Benzie and Strain^90^ with little modifications. Shortly, 10 μL of extracted samples and FRAP solution (190 μL) were mixed [composed of acetate buffer (300 mM, pH 3.6); FeCl 3.6H_2_O (20 mM) and TPTZ (10 mM); ratio 10:1:1 (v/v/v)]. Then the reaction mixtures were stored at 25 ± 1 °C for 15 min. Absorbance was recorded at wavelength (630 nm) using Microplate Reader (BioTek ELX800, Bio-Tek Instruments). Antioxidant capacity of samples was expressed in terms of TEAC (Trolox C equivalent antioxidant capacity).

- Antioxidant ABTS assay

To determine the ABTS antioxidant level, the proposed protocol of Tagliazucchi et al.^91^ was used with little modifications. Briefly, ABTS (2,2-azinobis (3-ethylbenzthiazoline-6-sulphonic acid) salt (7 mM) and 2.45 mM of potassium per sulphate were gently mixed in equal proportion to make ABTS solution, followed by incubation for 16 h in dark. The absorbance was measured at wavelength (734 nm), adjusted to 0.7 and solution then mixed with the extracts and incubated at 25 ± 1 °C in dark for 15 min. The absorbance of was recorded at 734 nm by using Microplate reader (ELX800, BioTek Instruments). Antioxidant capacity of samples was expressed in terms of TEAC.

### Quantitative (HPLC) determination of compounds

Detection and quantification were carried out via Varian HPLC system equipped with an online degasser (Metachem Degasit), an autosampler (Prostar 410) and a photodiode array detector (PDA, Prostar 335) using the protocol of Bourgeois, et al.^92^ and Wang, et al.^93^ with little modifications. The separation was performed at 35 °C on an RP-18 column (250 × 4.0 mm id, 5 µm; Purospher Merck). The mobile phase was composed of acetonitrile (solvent A) and 0.1% (v/v) formic acid acidified ultrapure water (solvent B). The composition of the mobile phase varied during a 1-h run according to a linear gradient ranging from a 5:95 (v/v) to 100:0 (v/v) mixture of solvents A and B, respectively, at a flow rate of 0.6 mL/min. Detection was performed at 260 nm for simple phenolics (gallic acid and caffeic acid), 360 nm for flavonoids (catechin, myricetin, kaempferol, isorhamnetin, and apigenin) and 210 nm for saponins (nahagenin, hederagenin, ursolic acid and betulinic acid). A typical HPLC chromatogram of a *F. indica* callus extract is shown in Supplemental Figure 1. Compounds were identified by comparison with authentic standards purchased from Sigma-Aldrich, and confirmed by standard additions and/or LC–ESI–MS analysis performed on Water 2695 Alliance coupled with a single quadrupole mass spectrometer ZQ. LC–ESI–MS data were collected in the positive and negative modes as described previously^1,2^. Data acquisition and processing were performed with MassLynx 4.0 software. All the compounds were quantified against 5-point calibration curves (R^2^ > 0.999) using authentic commercial standards (Sigma-Aldrich). The Quantifications were recorded using calibration curves and retention time of corresponding reference standards and the results were expressed as μg/mg DW of sample. 5-methoxyflavone (0.2 µg/mL) was used as internal standard for extraction (with detection set at 360 nm).

### Determination of anti-inflammatory activities

The callus cultures were also investigated for anti-inflammatory activities via employing sPLA2, 15-LOX, COX1 and COX2 assays by following the protocol reported by Usman et al.^94^ with slight modification.

- 15-LOX Inhibition Assay

The inhibitory activity in the extracts towards 15-LOX was also performed by the kit method (760700, Cayman Chem. Co., Interchim, Montluçon, France) according to following the manufacturer’s guidelines.

The amount of hydroperoxides accumulated thought the lipo-oxygenation reaction was calculated via the kit using standard filtered soybean 15-lipooxygenase in Tris–HCl bu_er (10 mM) at pH 7.4. The microplate reader (BioTek ELX800; BioTek Instruments, Colmar, France) was employed to measure the absorbance at 940 nm. The 5 µM Thioetheramide-PC was used as sPLA2 inhibitor. The extraction volume in equal amount was applied as blank.

- sPLA2 inhibition assay

The kit method (10004883, Cayman Chem. Co, Interchim, Montluçon, France) was followed with manufacturer instructions to measure inhibition of phospholipase A2 (sPLA2) enzyme. The Diheptanoyl thio-PC was used as substrate and thiotheramide-PC was applied as positive control inhibitor. While. Nordihydroguaiaretic acid (100 _M) was used as 15-LOX inhibitor during the assay. The extraction volume in equal amount was applied as blank. The absorbance was recorded via microplate reader (BioTek ELX800; BioTek Instruments, Colmar, France) at 420 nm.

The % inhibition was figured out with the following formula;

$$ \% {\text{ Inhibition }} = \, \left[ {\left( {{\text{IA}} - {\text{Inhibitor}}} \right)/{\text{IA}}} \right] \times {1}00 $$

where the inhibition is expressed as enzyme activity with adding inhibitor; IA as 100% activity of enzyme in the absence of inhibitor.

- COX-1 and COX-2 inhibition assay

The COX1 (ovine) and COX2 (human) assay kits were utilized by following the manufacturer guidelines (701050; Cayman Chem; Co, Interchim, Montluçon; France). The 10 µM ibuprofen as positive control and Arachidonic acid (1.1 mM) was employed as substrate. In order to estimate the activity, COX peroxidase component kit employed and Synergy II plate (BioTek Instruments, Colmar, France) was used to demonstrate the oxidized tetramethyl-p-phenylenediamine, Wurster’s blue (C10H16N2). The microplate reader (BioTek ELX800; BioTek Instruments, Colmar, France) was used to measure the absorbance at 5 nm for 5 min. The extraction volume in equal amount was applied as blank.

### Statistical analysis

All the experiments were performed at least in triplicates. Values represented as mean ± SE of at least three replicates. Origin 8.5 software (OriginLab, Northampton, MA, USA) was used to generate graphics with their mean data values and standard errors. The significance at *p* < 0.05 means and standard deviation were calculated by using Statistix 8.1 (Statistix, Tallahassee, FL, USA). All statistical analysis was performed with XL-STAT2019 (Addinsoft, Paris, France).

## Supplementary Information

Supplementary Infomation.
