# Supplementary material for: UV-C mediated accumulation of pharmacologically significant phytochemicals under light regimes in in vitro culture of Fagonia indica (L.)
Source: Sci Rep. 2021 Jan 12;11:679. doi: 10.1038/s41598-020-79896-6 (PMC7804141; doi:10.1038/s41598-020-79896-6)
Supplement: Supplementary file 1 — Supplementary Infomation. [file 41598_2020_79896_MOESM1_ESM.docx]

**UV-C mediated Accumulation of Pharmacologically Significant Phytochemicals under Light Regimes in *in vitro* culture of *Fagonia indica* (L.)**

Bilal Haider Abbasi^1, ∑,*^, Taimoor Khan^1, ∑^, Razia Khursheed^1^, Muhammad Nadeem^1^, Samantha Drouet^2, ∑^, Christophe Hano^2, ∑^

^1^Department of Biotechnology, Quaid-i-Azam University, Islamabad-45320, Pakistan

^2^Laboratoire de Biologie des Ligneux et des Grandes Cultures (LBLGC), INRA USC1328 Université ď Orléans 45067 Orléans Cedex2, France

*Correspondence: bhabbasi@qau.edu.pk


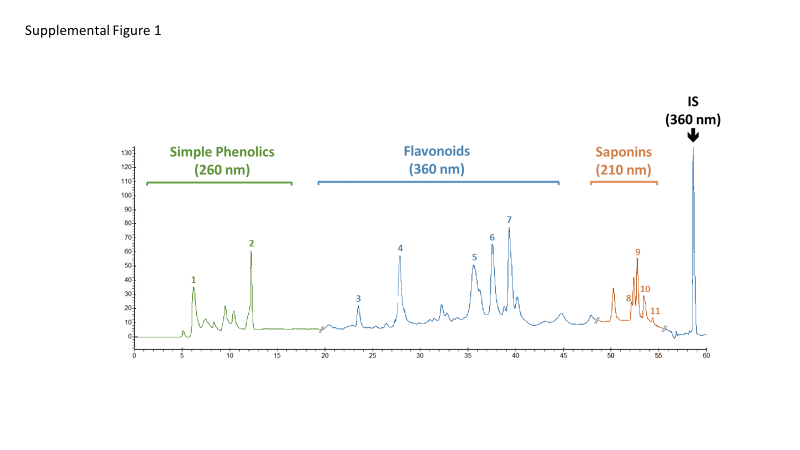


Supplemental Figure 1: Typical HPLC chromatogram showing the presence of simple phenolics (gallic acid (1) and caffeic acid (2) recorded at 260 nm), flavonoids (catechin (3), myricetin (4), kaempferol (5), isorhamnetin (6) and apigenin (7) recorded at 360 nm) and saponins (hederagenin (8), betulinic acid (9), ursolic acid (10) and nahagenin (11) recorded at 210 nm) in *in vitro* (callus) culture of *F. indica.* 5-methoxyflavone (0.2 µg/ml) was used as internal standard (detection set at 360 nm).

**Supplemental Table 1:** Pearson Coefficient Correlation (PPC) values between the main phytochemicals (gallic acid (GAL), caffeic acid (CAF), myricetin (MYR), catechin (CAT), kaempferol (KAE), isorhamnetin (ISO), apigenin (API), nahagenin (NAH), hederagenin (HED), ursolic acid (URS) and betulinic acid (BET) and antioxidant (FRAP and ABTS) and anti-inflammatory (COX1, COX2, 15-LOX and sPLA) activities.


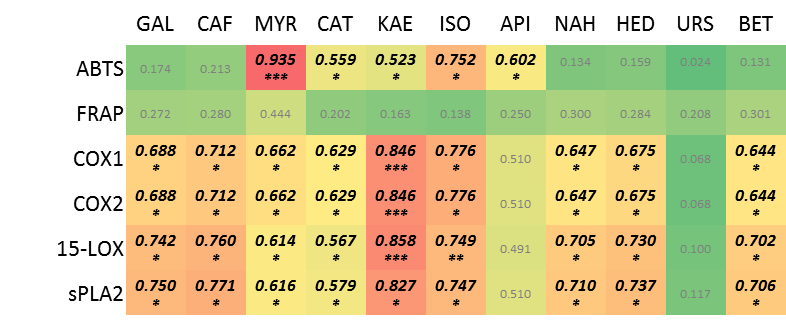


* for *p* < 0.05, ** for *p* < 0.01 and *** for *p* < 0.001
